# Soft and Hard Tissue Management in Implant Therapy—Part I: Surgical Concepts

**DOI:** 10.1155/2012/531202

**Published:** 2012-07-08

**Authors:** Antonio D'Addona, Marjan Ghassemian, Luca Raffaelli, Paolo Francesco Manicone

**Affiliations:** Institute of Clinical Dentistry, Catholic University of Sacred Heart, Largo A. Gemelli, 8 00168 Rome, Italy

## Abstract

Implant therapy has become a reliable and predictable treatment alternative for the replacement of missing teeth with conventional removable and fixed partial dentures. Recently though, in the pursuit for improved esthetics, the literature has dedicated a considerable amount of its research on the successful maintenance and regeneration of the surrounding gingiva and bone, which are lost following extraction of a tooth. Thoroughly analyzing the anatomic situation and well-planned treatment has become a requirement, because incorrectly planned and positioned implants may jeopardize long-term esthetic and functional prognosis. In addition, many types of biocompatible materials, autogenous hard and soft tissue grafts, and different surgical techniques have been developed, and their viability has been investigated. As a result, implant specialists have gained a greater understanding of the dynamics and anatomical and biological concepts of the periodontium and peri-implant tissues both at the surgical and prosthetic phases of treatment, which contributes to better soft and hard tissue management (SHTM). This may further contribute to achieving a superior final result which is obtained by having a harmonious soft tissue profile, a correctly placed and contoured final restoration, and the reestablishment of masticatory function and phonetics.

## 1. Introduction

The increasing demand over the years for highly esthetic results in all facets of dentistry has also influenced dental implants and has made achieving optimal esthetic results more challenging for the implant specialist and subsequently led to a greater consideration and study of all the contributing factors, both at the micro- and macroscopical level to achieve such a result. The challenge lies in the successful management and modeling of the papilla and gingiva, which are harmonious with the soft tissues of the adjacent natural dentition, and must also be maintainable long-term. Implant esthetics has been thoroughly studied [1–4], and several authors have proposed esthetic indices to assess peri-implant gingival tissues [5, 6] and implant crowns [7]. Belser et al. (2009) proposed the New Esthetic Index: Pink Esthetic Score (PES)/White Esthetic Score (WES) [8], a variation of previously introduced indices. 

Thus, an esthetically accepted result not only depends on the shade and form of the final restoration, but also in order to be achieved, it needs careful consideration, and often manipulation of the soft and hard structures adjacent to the implant, the abutment, and final restoration. This demand for better esthetics should accordingly alter the way in which implant specialists treatment plans and places dental implants, especially in the more esthetically demanding anterior region, by considering the soft and hard tissue management (SHTM) at the early treatment planning stage.

Part I of this paper will discuss the reason why surgical augmentation procedures are often required to enhance postextraction sites. Subsequently, it will discuss the use and selection of autogenous grafts, nonautogenous graft material, and finally the timing of implant placement in relation to the extraction, which have been identified as the key concepts in the SHTM. Part II of this paper will describe the key concepts both the theoretical and clinical prosthetic components which the literature has emphasized as having an important role in SHTM in implant therapy, by discussing their direct effect on these structures, and hence, on the final result.

## 2. Healing

**Figure 1:**
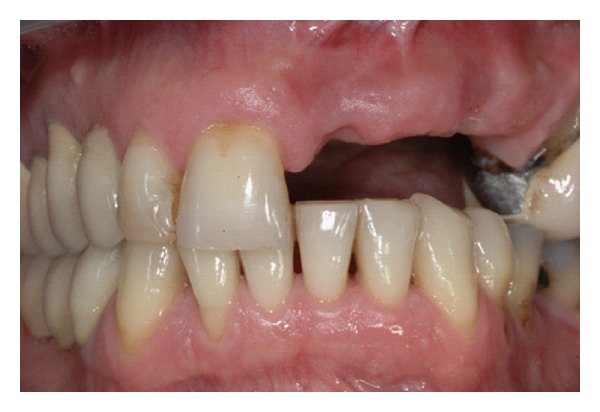
Frontal view of maxillary anterior zone: loss of vertical bone in the edentulous space with loss of papillae and altered profile of alveolar crest.

The periodontium is a dynamic complex of tissues [9] which undergoes remodeling in the area following extraction of a tooth, because the alveolar bone requires mechanical stimulation to maintain its form and density; hence, following the loss of a tooth, there is a decrease in trabeculation and loss of its height and width (Figure 1). This topic has been studied by many authors both microscopically and macroscopically, both in human models [10–13] and canine models [14] and it has been accepted that the remaining structural elements which undergo these changes are both the hard and soft tissues; they are dependant on each other.

In terms of soft tissue changes, immediately following extraction of a tooth, there is loss of gingival architecture, resulting in a reduction in the scalloped soft and hard tissue. The subsequent changes that take place involving these structures include the maturation of the wound which induces the formation and calcification of the bony material. From one of the earliest human studies conducted on the histologic events of the healing sockets [15], the authors concluded that at the first stage there is the formation of a clot, which is made up of a coagulum of red and white blood cells, fibrin, and inflammatory cells. At the second stage, the coagulum is replaced by granulation tissue over a 4-5-day period. At the third stage, over a 2-week period, the granulation tissue contracts and is replaced by connective tissue. This is followed by the fourth stage, in which the calcification of the osteoid at the base and periphery of the socket commences. This formation of bony trabeculae continues for about 6 weeks and is followed by the fifth stage, in which there is complete epithelial closure of the socket. By the 16th week, the socket is completely filled with bone and osteogenic activity is ceased [16].

 In results from a recent significant study carried out in human subjects [17], whereby the healing of the extraction socket was monitored for 6 months and analysis of cell populations was carried out, the authors indicated that there may be some differences to what previous research had suggested with regards to tissue healing following an extraction. This study has suggested that there is variability and possibly a delay in the formation and maturation of the alveolar socket following extraction of a tooth. It was found that the rate of healing varied significantly between subjects and that the process of replacement of woven bone with lamellar bone and marrow was slow, such that bone organization and architecture were not completed at 24 weeks after tooth extraction. This study is in contrast with earlier studies carried out and its clinical implications will be discussed subsequently.

In terms of the surrounding hard tissues, research has shown that once the tooth has been extracted the alveolar bone underwent resorption. This dimensional change has been measured both qualitatively, in terms of the surfaces which undergo the resorption, and quantitatively over a period of time and identified as loss in alveolar bone in both the buccolingual and vertical dimensions, resulting in a reduced gingival profile [11, 13, 18], especially if it is of a thin biotype. At a macroscopical level, the healing results in changes in both the bone and overlying soft tissues. Most of the bone resorption which takes place is in a buccolingual dimension, since there is about a 5–7 mm reduction in the alveolar bone crest, over a 6–12-month period, though most of the reduction takes place in the first 4 months. Concurrently, there is a reduction in the vertical dimension of the alveolar bone of approximately 2–4.5 mm. The resorption which takes place may be increased in sockets of molars and in multiple adjacent extraction sockets [13].

Significant hard tissue changes following tooth extraction and implant placement have also been observed in animal studies, including surface resorption of the buccal and lingual walls, the resorption of the marginal bundle bone, and reduction in height of the buccal bone, and reported that as healing continued the height of the buccal wall continued to resorb [19, 20]. It was also found that the buccal plate of the alveolar was often thinner than the lingual plate, and that in these cases this also was associated with more resorption [21, 22].

Other factors that can cause further bone resorption are a reduced alveolar bone width and injury to the alveolus, which may be sustained before or during tooth extraction as iatrogenic fracture. Other local factors include pathology caused by any infective process, such as periodontal and endodontic abscesses, cysts, and tumors [11]. The rate and type of resorption may be increased due to the formation of a fibrous tissue in the damaged areas, which may prevent normal healing and osseous regeneration from taking place [13]. Systemic conditions which may induce further bone loss include genetic predisposition, general and medical conditions, such as diabetes and smoking, and medication and bisphosphonates use [23].

## 3. Autogenous Grafts

The most common problem the implant specialist is often faced with is the lack of sufficient bone, both the vertical and horizontal dimensions, which if not contemplated at the initial stages of treatment, will induce an esthetically and functionally unacceptable result. From the literature, many authors have approached the problem of alveolar atrophy by proposing different combinations of surgical techniques and procedures to replace or augment the defective tissue, and from this research have evolved many procedures, methodologies, and materials to encourage new tissue formation, or to discourage further loss of tissue following extraction of dental elements. Various grafting procedures have been developed, using autogenous bone grafts from various donor sites, which has been set as the gold standard for bone augmentation. Autogenous bone can be augmented in particulates or as a block depending on the amount of bone which has been lost and will need to be regenerated.

An autogenous graft is the gold standard because being patient's own bone there are many advantages to its use [24–26]. They contain live osteoblasts and osteoprogenitor cells, which proliferate and bridge the gap between the graft and recipient bone. Success rates are high because there is no immune reaction and the microscopic architecture is perfectly matched. Autografts usually result in the greatest regeneration of missing bone, due to minimal postoperative resorption of the grafted bone. In most cases, the acceptable donor site for block grafts is found intraorally and often enough in proximity to the area to be regenerated.

## 4. Origin of Graft

In earlier study carried out by Ozaki et al. [27] on animal subjects, the authors investigated the possible influence of microarchitecture and embryologic origin as influencing constituent for the success of autogenous onlay block grafts in the craniofacial skeleton. Prior to this study, it was believed that the embryologic origin of the graft is the main influencing factor, because grafts of membranous origin performed better than endochondral grafts. Though it remains a contributing factor for its success. From this study, the authors concluded that the microarchitecture was the more important constituent in the maintenance of onlay block grafts, and in particular cortical block grafts performed better than cancellous bone grafts.

Comparisons have also been made to determine the most successful source of autogenous graft, using more specific human study on bone augmentation procedures. In one study, 46 successful implants were placed in 32 patients who had the implants sites using 3 different autogenous donor sites. It was concluded that each of the donor sites resulted in a certain amount of bone regeneration, but between the groups significant differences were noted. The group that exhibited the most amount of bone regeneration was the group with the autogenous graft taken from the mandibular symphysis, followed by the ramus and finally the maxillary tuberosity. This study confirmed the success of these augmentation techniques and successfully identified the most reliable donor site in terms of bone gain [28]. And once again, this is in agreement with our clinical experience as the symphysis and ramus are the preferred donor sites.

The occurrence of complications and morbidity between ramus and symphysis have been compared in a retrospective human clinical study, whereby patients were asked to complete a questionnaire, followed by a clinical examination for other signs and symptoms such as sensory impairment [29]. From this study, the authors concluded that the ramus was preferred as a donor site for autogenous block grafts after modifying the surgical technique and increasing the access to the mandibular body area and using a long-shafted bur to create a groove instead of an inferior border osteotomy. Even though the symphysis grafts are more accessible, the ramus grafts encounter fewer complications and morbidity.

Another interesting aspect of onlay block grafts has been studied in a recent study, whereby authors compared the biotype of the patients in relation to the successful maintenance of the block grafts. A two-stage approach was used for implant placement in 40 patients who were categorized as either having a thin or thick biotype and the regenerated bone site was analyzed using computerized tomography for an average of 3.5 years. From their results, authors concluded autogenous block grafts can be used to restore both function and esthetics predictably and that the biotype of the teeth adjacent to the implant sites did not have an influence on the maintenance of the volume of the block grafts [30].

## 5. Surgical Graft Procedure

The viability of bone regenerated in autogenous guided bone regeneration (GBR) procedure has been thoroughly researched. In one study, the bone augmentation was carried out with autogenous block graft and nonresorbable barrier membranes. Of the 66 implants placed in regenerated bone sites, 60 were concluded to be successful, and the authors concluded from their clinical results that implants placed in generated bone using this particular technique were comparable to the results which can be achieved in nonregenerated bone [31]. This conclusion has been confirmed by other similar studies [32, 33], on the success of these GBR techniques.

The use of autogenous bone grafts used for vertical ridge augmentation can also be carried out with either resorbable barriers or nonresorbable titanium-reinforced barriers. In another study carried out where these two vertical bone augmentation procedures are investigated and compared, 22 implants were placed, half were assigned to each group. Though the number of implants placed was too small, the results indicate that no significant difference in bone gain was present between either of the procedures, and thus the authors concluded that both GBR techniques were effective for vertical bone augmentation [34].

**Figure 2:**
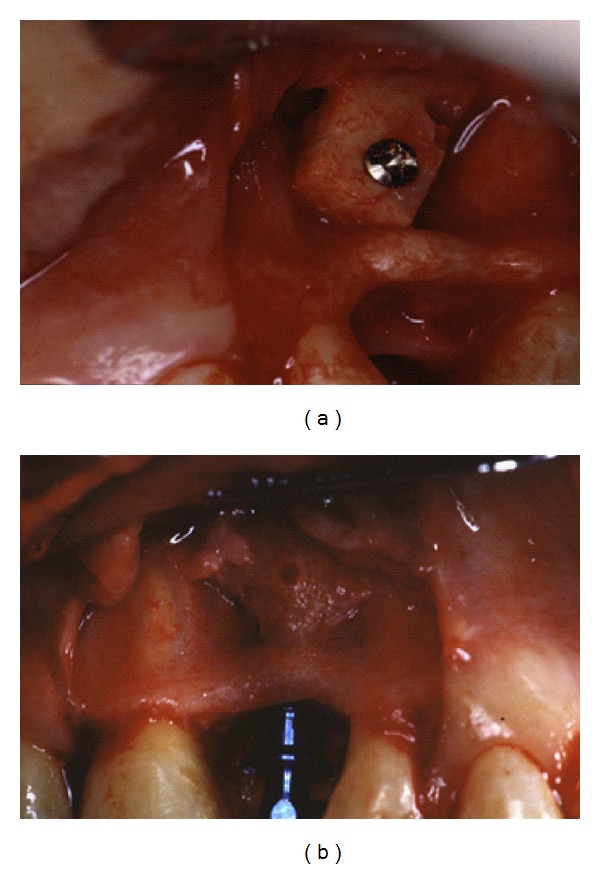
Intraoral view of autogenous block graft taken from the mandibular symphysis, at time of fixation in no. 24 site (a). Intraoral view of placement of implant in no. 24 site after successful graft, at time of removal of fixation screw (b).

The use of either resorbable or nonresorbable membranes, however, has been associated with a greater number of postoperative complication such as exposure of the membrane, and infection of the surgical site [34]. Conclusions from a clinical study where autogenous cortical block grafts were used without the use of membranes, but were fixated with titanium screws indicate that this technique is safe, effective, and simple [35]. This was confirmed by other human studies, one comparing the effectiveness of augmentation of bone using autogenous block grafts and osteodistraction [36], and the other using autogenous block grafts without membranes [37]. In our continuing experience, autogenous block grafts fixated without membranes is a valid treatment option for bone augmentation prior to implant placement. This is due to the cellular and microarchitectural events following the positioning of an autogenous graft, which confirm its osteoinductive and osteogenetic characteristics [38] (Figure 2).

## 6. Nonautogenous Graft Material

The most commonly used nonautogenous materials are demineralized freeze-dried bone. When first introduced, they were used for augmentation in periodontal defects. Its bone forming properties are described as osteoconductive and slightly osteoinductive and its use has proven to be effective in periodontal regeneration [39]. Subsequently, its use was extended to socket preservation [40, 41] and implant therapy where augmentation procedures were indicated [42]. This nonautogenous material has many different applications, as it can be used in combination with autogenous bone grafts and resorbable and nonresorbable membranes. More recent human studies confirmed the efficacy of demineralized freeze-dried bone in augmentation procedures combined with implant therapy [43–45]. Authors from a recent retrospective radiographic study, whereby demineralized freeze-died bone was used in post-extraction sockets exhibited similar marginal bone loss to implants placed in patients bone [46].

Another more recent nonautogenous graft material is hydroxyapatite of *β*-tricalcium derivatives. This material alone does not have bone-forming capabilities and must be used in combination with other autogenous or nonautogenous graft materials. It shows elevated bioactive and biocompatible characteristics in physiological conditions because of the similarities it has with the inorganic components of bone [47]. This synthetic material has been extensively researched in animals [48–53]. From a recent animal study, the authors concluded that this new synthetic bioresorbable material scaffold may be potentially used in lateral ridge augmentation, though further long-term experimentation with regards to surgical technique and human studies is necessary [54].

Both these nonautogenous materials are used to augment hard tissue. However, a potentially significant preliminary report has been published with regards to peri-implant soft tissue augmentation. In this study of 6 patients who had previously had bone regeneration procedures, the authors used a resorbable collagen matrix as a scaffold for human platelet-derived growth factor. A moderate increase in soft tissue was found, however, authors concluded that improved measuring techniques were required to accurately measure soft tissue changes in volume [55]. 

## 7. Surgical Timing

### 7.1. Timing between Extraction and Implant Placement

The timing between extraction of a tooth and placement of an implant is an important factor in determining the esthetic and functional success of the final restoration, because it can indicate to the implant specialist the amount of bone resorption and loss of the soft tissue profile, which may have taken place within this time. Since it has been known that the rate of alveolar bone resorption is greatest in the first year after extraction, implant specialists try, if possible, to place the implants before a significant amount of resorption takes place. Relative to the postextractive time, the timing of the implant placement has been subdivided into three groups: immediate, delayed, and staged. In the immediate group, the implant placement occurs at the time of the extraction in the delayed group, the implant is placed approximately 2 months after the extraction, to allow complete soft tissue healing and closure of the socket. Whereas in the staged group, the placement of the implant is carried out about 6 months following the extraction, to consent a significant amount of bone healing [56].

### 7.2. Immediate Placement

The advantages claimed for the immediate implant placement protocol are the marked reduction in time taken for healing, the reduced number of surgical procedures, and the optimal availability of existing bone to allow primary stability of the implant. Furthermore, at a microscopical level, it is thought that the postextraction osteogenic activity may improve the bone-to-implant contact when surface-treated implants are used [57]. Conclusions from another study which examined hard tissue changes following immediate implant placement only, partially supported the fact that bone defects around immediate implants could heal. The authors indicated that though from clinical examination new bone formation was observed, at a microscopical level the presence of a connective tissue layer was suspected, thus, they could not draw conclusions as to whether in these sites osseointegration between the bone and implant surface took place [58]. In addition, the buccolingual positioning of immediate implants must be carefully considered, because in contrast to what was previously thought, the immediate implant protocol does not completely prevent the buccolingual resorption of the buccal plate [59].

Another conceivable disadvantage of this technique is that it requires an acceptable amount of bone tissue, since it does not permit large amounts of hard and soft tissues to be grafted or augmented. If there are significant pathologies affecting the hard and soft tissues in the area of the extracted tooth, this technique is not recommended [13]. Some research on immediate implant placement has indicated that bone remodeling, apposition, and healing of bony tissue take place also in the implant neck area. This is believed to be the reason for the lack of implications which negatively affect the final esthetics [60], and conclusions from a 1-year clinical study whereby 35 immediate implants were placed have also confirmed that the immediate implant placement protocol can result in satisfactory peri-implant soft tissue and esthetic outcomes [61].

A recent review has been carried out examining the clinical outcomes of immediate or early placement protocols. The conclusions made from this literature analysis suggest that the use of the immediate procedure can result in high success in terms of survival rates. Whereas in terms of esthetic outcome, the authors did not make a decisive statement but concluded there is an increased risk of esthetic failure, though it is believed that if a thorough process of case selection is carried out, satisfactory results can be achieved. Therefore, the authors have suggested that these protocols can be used by implant specialists with an elevated level of experience [62]. This is in agreement with our clinical experience which underlines that no ideal timing exists between extraction of a tooth and implant placement, as each patient should be evaluated case by case. Though as a general rule, immediate implant placement protocol may be used in the posterior segments, whilst the delayed protocol is still preferred in the anterior segments.

### 7.3. Delayed Placement

As well as permitting primary soft tissue closure, the rationale to the delayed implant placement technique is to allow for the resolution of all signs of minor pathology associated with the tissues in the area of the extracted tooth, and to consent complete bone healing, thus, optimizing the healing and osseointegration of the implant, and integration of the tissues, if bone augmentation procedures and soft tissue grafts have been carried out [21].

As discussed earlier, one particular study carried out a comparative analysis to evaluate the healing of buccal marginal defects around implants after one year of being placed directly in extraction sockets (immediate), or 4–6 weeks (delayed immediate) after extraction and augmentation procedures with membranes and bone grafts. In relation to the timing, in the case of single implants, better statistical results were found with the delayed immediate protocol, due to the primary closure of the alveolus which seemed to help the integration of the implant, bone grafts, and barrier membranes [20]. These results were confirmed in another similar study that was carried out to compare the immediate and delayed protocols. However, in terms of the dimensions of the interproximal papilla, the authors were unable to significantly differentiate between the groups [63].

### 7.4. Staged Placement

When compared to the immediate technique of implant placement, it can be more difficult to achieve highly esthetic restoration with the staged technique, because of the resorption of the alveolar bone, which potentially results in a narrower ridge with a buccal concavity and contemporarily the loss of gingival architecture. In these cases, as it has been previously discussed, the implant specialist should decide to carry out soft and hard tissue regeneration procedures based on radiographic diagnosis and prosthetic treatment planning to restore the lost tissue and achieve satisfactory esthetics. Therefore, disadvantages of this technique include longer treatment time and increased number of surgical interventions.

## 8. Conclusion

It is imperative that implant specialists evaluate the presenting condition of each case individually, and carefully consider the consequences of the surgical interventions and their timing, to be able to achieve an acceptable result. Based on the initial condition of the hard and soft architecture, implant specialists must decide firstly whether hard or soft tissue augmentation are necessary prior to implant placement, and if so, which technique is appropriate. From the literature and based on our experience, autogenous bone grafts are a viable treatment option for hard tissue augmentation when there is not sufficient bone and particularly the use of block grafts taken from the mandibular symphysis area or the ramus, when a large quantity of graft material is required. In relation to timing-for-implant placement, our protocol follows the literature guidelines and the decision should be made for each case on an individual basis.
